# Miscellaneous Facts and Observations

**Published:** 1783

**Authors:** 


### SECTION II.

### ESSAYS AND OBSERVATIONS.

I. *Miſcellaneous Facts and Obſervations, communicated in a letter to* Dr. Simmons, F. R. S. *by* Thomas

Thomas Percival, M. D. F. R. S. *and* S. A.
*Member of the Royal Medical Society at Paris,* &c.

THE great Lord Verulam recommends the collection of facts, experiments, and obfervations, as the beft method of promoting the improvement of Phyfic; and in his effays he exprefsly confines himfelf to "certain brief "notes, fet down rather fignificantly, than "curioufly." This mode of writing is confonant to the plan of the Medical Journal, and is often more agreeable to men of letters, than long and fyftematic compofitions: for it precludes the labour of reading or of repeating elementary propofitions, and well-known truths; renders the detection of error more eafy; facilitates the communication of new difcoveries; and prefents to the mind, in feparate and diftinct views, the real additions which are made to fcience*. How far the influence of thefe confiderations, or the fanction of the high authority I have quoted, may juftify the following mifcellaneous remarks, I fhall leave to your decifion.

* See the Author's Preface to Philofophical, Medical, and Experimental Effays.

I. *Retrograde motion of the Lymphatics.*

A very ingenious young phyſician and philoſopher, who, had he lived, would have done honor to his profeſſion, has advanced a variety of arguments to evince a nearer communication between the alimentary canal and the bladder*, than by the ſanguiferous circulation. The following caſes may, perhaps, illuſtrate and confirm this doctrine.

Mrs. W—— had laboured under a *diarrhœa* many weeks, when I was called to her aſſiſtance on the 13th of June 1780. I directed for her the following pills:

℞. Extract. ligni Campech. gr. v.

Philon. Londinenſ. gr. x.

Pulv. ipecac. gr. j.

Syrup. Cydon. q. ſ.

M. f. pil. iij. ter die ſumendæ.

By the uſe of theſe pills her diſorder ſoon abated.

June 30. She complained of nauſea, heat, and thirſt. The pulſe was not increaſed in velocity, and ſhe was ſtill inclined to be lax. The following mixture was preſcribed:

* See Mr. Charles Darwin's Experiments, &c. Litchfield, printed 1780.

℞. Aq.

℞. Aq. cinnam. ten. ℥vij.

Extract. ligni Campech. ʒj.

Sal. tart. ℈iv.

Syrup. Cydon. ʒiij.

M. cap. ℥jſs ter die in effervefcentia cum cochleare amplo fucci limonum.

This remedy feemed to obviate all her complaints. But in the morning of July the 5th fhe had a fudden and urgent call to difcharge her urine; and after voiding treble the ufual quantity, fhe obferved, that it was of a pink colour, inclining to purple, very fimilar to that of the medicine which fhe had taken. I found her under fome alarm on account of this circumftance; and examining her water in a glafs, I was fatisfied that its hue was different from that of blood: and from the appearance I concluded, that it was ftrongly tinctured with logwood. In this opinion I was afterwards confirmed by its tafte, which was fweetifh and fub-aftringent; and by the deep purple it affumed, when a fmall portion of the falt of fteel was added to it. The urine of this patient had not, during the previous courfe of her illnefs, varied from its natural colour, although eight fcruples of the *extractum ligni Campechenfis* had been adminiftered. About an hour before the prefent

 fingular

ſingular evacuation, ſhe had taken the uſual doſe of her medicine; yet in the evening of the ſame day, her urine exhibited no marks of the logwood.

In this caſe there appears to have been a ſudden and copious tranſlation of urine into the bladder, probably by means of the retrograde motion of the lymphatics: and the fluids, conveyed with the lacteals, were ſtrongly imbued with a medicine, which, together with its colouring particles, evidently retained alſo its aſtringent quality *.

September 27th 1780. Mrs. C—— had been ill more than two months of a pulmonary conſumption, which was accompanied both with colliquative ſweats and a *diarrhœa*, when I was conſulted by her. I preſcribed a gentle emetic, and afterwards the following draughts:

℞. Emulſ. commun. ℥j.

Myrrh. mucilag. gum. arab. ſolut. gr. xij.

Elix. pareg.

Sp. nit dulc. aa. ʒſs.

Sal. tart. ℈j.

* Dr. Lewis, in his excellent Materia Medica, has obſerved, that logwood ſometimes colours the urine, but he does not ſeem to have been apprized, that it communicates aſtringency to it.

Succi.

Succi limonum ℥ſs.

M. f. hauſt. ter die in effervescentia ſumend.

Her cough was alleviated, and the ſweating and *diarrhœa* almoſt entirely reſtrained by theſe draughts. But during the uſe of them all the urine, which ſhe voided, had the colour of a dilute ſolution of logwood.

October 3d. The *diarrhœa* returned, and I had again recourſe to *extractum ligni Campechenſis*, which was now given every fourth hour, in doſes of fifteen grains, diſſolved in ſimple cinnamon water. The purging was ſoon checked, and the urine aſſumed the ſame pinky colour as before. A portion of it was turned black by immerſing in it the blade of a table knife: nor did the excretion change its appearance till the extract of logwood was laid aſide.

This inſtance of the *uniform* and *permanent* conveyance of a colouring aſtrictive matter to the bladder, may perhaps ſeem leſs favourable to the doctrine of a retrograde motion of the lymphatics, than the caſe before recited. But is it probable that ſo large a quantity of logwood, retaining its peculiar and active properties, could have been introduced into the blood veſſels, without occaſioning ſome unuſual ſymptoms? Or

Or could the urine, in so short a space of time, have been replete with it, if it had been previously mixed with the whole mass of circulating fluids? Be this, however, as it may, it is a curious and interesting fact, that a vegetable astringent is capable of surmounting the powers of digestion, of passing into the absorbent vessels, and of being carried unchanged to the urinary organs. Various practical uses may possibly be founded upon it; and it may furnish a juster theory of the action of the Peruvian bark, in glandular tumours, and disorders of the lymphatic system. Certain it is, that this very efficacious medicine is remarkably retentive of its virtues. Fuller says, with some degree of admiration, *Cum olim, experimenti causa, ejusdem (corticis) decoxissem, non eo usque vires ejus exhaurire valui, quin vel octavum decoctum adhuc amaricaret.* If his patience had permitted him to extend the experiment, he would have found, as I have done*, that even twenty-five coctions, and thirty cold macerations, are insufficient to exhaust its virtues.

It is well known, that madder, taken internally, tinges the urine red; and that it produces a similar effect even upon the bones of animals,

* Essays, Medical and Experiment. vol. I.

though though neither the flefhy nor cartilaginous parts of the body fuffer any alteration by its ufe. Nor will the bones, when thus ftained, yield any colour to water or fpirit of wine. The root of this vegetable has a bitterifh, and fomewhat auftere tafte; but I have not yet afcertained, by any experiment, whether it impregnates the urine with an aftrictive quality. The colouring matter of rhubarb is fpeedily conveyed to the bladder; but the water thus imbued did not, in a late trial, exhibit the flighteft appearance of purple, on mixing with it a fmall quantity of the falt of fteel.

Little attention has hitherto been paid to the change of quality produced, by food or medicine, on the fecretions and excretions of the human body, although very ufeful information might be derived from it. Thus, for example, could we medicate the milk of nurfes, we fhould be better qualified to cure the difeafes of the children whom they fuckle. There is a fpecies of fœtid breath *(dyfodia pulmonica)*, to which perfons of a narrow cheft and fcorbutic habit are peculiarly incident. It feems to originate from the want of power to make a full expiration, by which too much perfpirable matter is retained, and corrupted by ftagnation in the vesicles of the lungs. In such cases I have found the most salutary effects from the use of myrrh and fixed air, internally administered. These sweetening and antiseptic substances are probably carried to the lungs, and discharged together with the offensive vapour, which they correct, at the same time that they invigorate the smallest ramifications of the bronchiæ. For I cannot impute their action, *solely* at least, to their corroborant powers; because neither steel, the Peruvian bark, nor other tonics, are exhibited with the same success.

It has been shewn above, that an astringent substance may be conveyed to the bladder, so as strongly to impregnate the urine. And there is no reason to presume, that this is owing to any particular subtlety in the *lignum Campechense*. Were proper tests to be applied, the presence of other remedies might, perhaps, be frequently discovered in that recrementitious fluid. And thus we should have at command various means of cure, adapted to the disorders of those organs. In another work I have given a decisive proof, * that by drinking copiously of mephitic water, the urine may be almost saturated with fixed air.

* Philosophical, Med. and Experiment. Essays.

This

This appeared from the precipitation which ſuch urine produced in lime water, from the bubbles which it emitted, and from the ſolution of ſeveral *calculi* that were immerſed in it. And hence we have acquired the practical knowledge of a ſafe and efficacious diſſolvent, both of the ſtone and gravel.

I ſhall cloſe this ſection with a paſſage from Dr. Fuller, which, if it be the reſult of accurate obſervation, corroborates what has been above advanced. Speaking of the balſam of Copaiba, he ſays, "*Sapore donatur amaro, acre, terebinthinaceo, admodum penetranti, et in ore durabili; atque licet videatur eſſe quædam terebinthinæ ſpecies, urinam tamen odore violaceo minime inficit; illam vero ſapore amaro imbuit, ejuſque et ſeri ſanguinis, et ſalivæ muriaticam ſalſedinem mirifice delet.*"

### II. *Reciprocal Sympathy between the Stomach and the Lungs.*

A Gentleman, afflicted with a purulent expectoration, has found, by repeated experience, that his fits of coughing may often be ſuppreſſed by a draught of cold water, or a ſmall glaſs of wine. His courſe of life has been intemperate; and theſe remedies, by diminiſhing the *atonia* of the ſtomach, and producing a grateful ſenſation in in that organ, allay, for a while, the irritation which ſubſiſts in the lungs. In ſuch caſes of phthiſis, which frequently occur, the exhibition of nitre, or other cold debilitating medicines, tends to aggravate the diſeaſe, and to haſten its fatal termination. To patients, under theſe circumſtances, porter is generally a very agreeable and ſalutary beverage. It quenches thirſt; quiets the cough; checks the ſweating; and, if drank only in ſmall quantities at once, does not accelerate the pulſe, or augment the hectic fever.

Ann Ogden, aged ten years, was admitted an out-patient of the Mancheſter infirmary, and put under my care on the 22d of November 1779. On the 18th ſhe had been attacked with a moſt pungent pain in the ſtomach. The ſucceeding day, a violent ſpaſmodic cough enſued, and her ſtools were obſerved to be bloody. She was ſoon relieved by opiates, mild purgatives, and a ſoft demulcent diet: for the ſwallowing of a pin gave riſe to theſe complaints; and the progreſs of them clearly evinces the ſympathy which the lungs have with the ſtomach. To this law of the animal œconomy, phyſicians and phyſiologiſts have paid ſufficient attention; but they have not equally noticed the reverſe of it; though it is no leſs certain, that various pulmonic affections affections may powerfully influence the ſtate of the ſtomach; and that the conſequent ſymptoms furniſh very important indications of cure.

A phyſician conſulted me, in February 1780, on account of a ſevere aſthma, of the humoral kind, to which he was ſubject. At the commencement of the diſeaſe, he could take ſeveral drachms of the *vinum ipecacoanhæ*. But as his diſorder increaſed, the irritability of his ſtomach became ſo great, that fifteen drops of the ſame wine often acted as an emetic. The medicine affording conſiderable eaſe to his breathing, he gradually augmented the doſe of it as he grew better, till he could bear a drachm or two without retching, and almoſt without nauſea.

Mr. ———, in a ſevere peripneumony, took, every fourth hour, two ounces of a decoction of ſeneka root and liquorice *. The remedy created no uneaſineſs, and ſeemed to give relief, whilſt the patient was in an erect poſture; but when he lay down, a poſition which rendered his

* The diſagreeable ſenſation, produced by the Seneka root, in the fauces, is much abated by combining with it, in decoction, a ſufficient quantity either of the root or the extract of liquorice, the demulcent quality of which is an excellent auxiliary in the peripneumony.

breathing more difficult, every dofe of it aggravated the dyfpnœa. In this inftance there feems to have been a reciprocal, or interchangeable action between the organs of digeftion and of refpiration. The increafe of dyfpnœa produced an increafe of irritability in the ftomach; and the feneka root, under thefe circumftances, proved fo ftimulant as to aggravate the oppreffion of the lungs. This cafe, and I could cite many others, affords a ftrong confirmation of the propriety of Sydenham's practice, in keeping his peripneumonic and pleuretic patients out of bed as much as their ftrength would permit.

### III. *Dyfury.*

Mr. —— of Knutsford, applied to me in January 1782 for advice concerning an arthritic vertigo. I directed a blifter to the nape of his neck, and the following pills:

℞. Gum. guaiac. ʒij.

Sal. ammon. vol. ʒj.

Balfam guaiac. q. f.

M. f. pil. medioc. cap. iij. ter de die.

A violent ftrangury enfued. The medicine was difcontinued; the blifter removed; and this painful complaint foon ceafed. In a few days the pills were repeated; but the firft dofe renewed the the affection of the urinary organs. A drachm of the *species aromaticæ* was then substituted in lieu of the volatile salt; and this formulary occasioned no pain or inconvenience. But having more confidence in the efficacy of the pills first prescribed, I advised the further trial of them at the end of ten days: they produced, however, a third time the symptoms of strangury, and were therefore entirely laid aside. Is it not probable, that in this case there was a translation of the gouty affection to the urinary passages, produced by the blister, and augmented by the stimulus of the volatile alkali? A predisposition, being thus formed in those parts, the volatile salt alone proved afterwards sufficient to renew the malady.

There is a species of chronic dysury, to which persons of an arthritic or scorbutic habit, and who have passed the meridian of life, are peculiarly incident. It is often mistaken for the stone, and aggravated by the use of lithontriptics. Indeed it has many symptoms in common with that disorder; such as frequent and urgent calls to make water; pain at each extremity of the urethra; a mucous discharge; tenesmus; and sometimes a suppression of urine. But the patients, who labour under it, feel no uneasy weight weight in the perinæum, and always void their water with much lefs difficulty in an erect than in an horizontal pofture. The complaint, alfo, may be further diftinguifhed from the ftone by having fhorter intervals of eafe; by more frequently injuring the retentive power of the bladder, and by occafioning no fudden interruption to the ftream of urine in the abfence of pain. It feems to arife from an acrid defluxion on the internal coat of the bladder, which is thereby rendered fo exquifitely fenfible, that the ftimulus of the urine becomes almoft intolerable, and very frequent efforts are excited to expel it: thefe efforts, however, fhould be reftrained as much as poffible, becaufe they tend to increafe the pain and irritation of the bladder, and to prevent the complete difcharge of its contents; for that organ cannot effectually contract itfelf without a due degree of previous diftenfion.

I have tried various remedies in this diforder, but have found none fo fuccefsful as mercury, which feldom fails to afford relief, and generally produces a cure, if adminiftered with perfeverance and in fufficient quantity. According to the urgency of the cafe, one, two, or three fcruples of the *unguentum cæruleum fortius* fhould be rubbed into the thighs every night, till a flight

ſlight ptyaliſm enſues: the ſymptoms for the moſt part abate before the ſpitting comes on, and after it has continued a-while, they diſappear entirely.

I was firſt induced to adopt this mode of treatment from my experience of the ſalutary operation of the remedy recommended by Dr. Gilchriſt, in a diſorder of the bladder, which bears ſome analogy to that which I have deſcribed*; but having found that the mercurial pill is apt to diſturb the bowels, and conſequently that it is leſs certain of admiſſion into the ſyſtem, I have in my later practice preferred the uſe of the *unguentum cœruleum*. In ſlighter caſes, indeed, I ſometimes give half a grain of calomel, with two grains of James's fever powder, twice every day; and this ſmall doſe of mercury, if duly continued, may ſuffice to effect a cure, without producing any ſalivation, or even ſoreneſs of the mouth. In a late inſtance, an habitual head-ach, with which a difficulty and pain in making water were complicated, gave way to this remedy.

From the ſalutary operation of mercury in the dyſury, it may be ſuſpected, perhaps, that

* Phyſical and Literary Eſſays, vol. iii.

the the difeafe originated from the lues venerea. I formerly entertained this idea myfelf; but further experience has convinced me, that it has no foundation in more than half the cafes which occur; and confequently, in explaining the action of the remedy prefcribed, we muft not have recourfe to the fecret powers of a fpecific or an antidote.

