# Errata

**Published:** 1783

**Authors:** 


					p. 60, to the iafc
lint of the frcjcripticn add Extract:. Lign. Campcch. gjr* xv. j

				

